# Supplementary material for: Are Household Expenditures on Food Groups Associated with Children’s Future Heights in Ethiopia, India, Peru, and Vietnam?
Source: Int J Environ Res Public Health. 2020 Jul 1;17(13):4739. doi: 10.3390/ijerph17134739 (PMC7370180; doi:10.3390/ijerph17134739)
Supplement: Supplementary file 1 [file ijerph-17-04739-s001.pdf]

International Journal of
Environmental Research and Public Health

Table S1. Unadjusted and Adjusted Models for Predicting Child HAZ Using Past Household Starches Expenditures in Ethiopia.

**Ethiopia—Expenditures at 5 years Predicting HAZ at 8 years**										
**Starches**	**Model 1**		**Model 2**		**Model 3**		**Model 4**		**Model 5**	
**Model Adjusted R-Squared**	**0.0123**		**0.0170**		**0.0484**		**0.052**		**0.0625**	
	**β**	***p*-Value**	**β**	***p*-Value**	**β**	***p*-Value**	**β**	***p*-Value**	**β**	***p*-Value**
Starches	0.02	<0.001	0.02	<0.001	0.02	<0.001	0.004	0.414	0.004	0.460
Individual Variable										
Female			0.14	0.003	0.13	0.005	0.13	0.004	0.13	0.006
Community Variable										
Rural/Urban Status					−0.35	<0.001	−0.33	<0.001	−0.20	<0.001
Household Variable										
Total Food Expenditures							0.01	0.007	0.01	0.062
Maternal schooling										
No Schooling									Reference	
1–6 years									0.14	0.016
7–12 years									0.21	0.014
12+ years									−0.001	0.997
Paternal schooling										
No Schooling									Reference	
1–6 years									0.15	0.013
7–12 years									0.20	0.013
12+ years									0.28	0.027
**Ethiopia—Expenditures at 8 years Predicting HAZ at 12 years**										
**Starches**	**Model 1**		**Model 2**		**Model 3**		**Model 4**		**Model 5**	
**Model Adjusted R-Squared**	**0.0066**		**0.0060**		**0.0505**		**0.0509**		**0.0580**	
	**β**	***p*-Value**	**β**	***p*-Value**	**β**	***p*-Value**	**β**	***p*-Value**	**β**	***p*-Value**
Starches	0.01	<0.001	0.01	<0.001	0.01	0.009	0.003	0.592	0.01	0.320
Individual Variable										
Female			0.01	0.908	−0.01	0.844	−0.01	0.845	−0.02	0.704
Community Variable										
Rural/Urban Status					−0.42	<0.001	−0.41	<0.001	−0.30	<0.001
Household Variable										
Total Food Expenditures							0.004	0.206	0.001	0.782
Maternal schooling										
No Schooling									Reference	
1–6 years									0.15	0.007
7–12 years									0.19	0.029
12+ years									0.13	0.478
Paternal schooling										
No Schooling									Reference	
1–6 years									0.09	0.119
7–12 years									0.13	0.099
12+ years									0.16	0.193

Table S2. Unadjusted and Adjusted Models for Predicting Child HAZ Using Past Household Protein Expenditures in Ethiopia.

**Ethiopia—Expenditures at 5 years Predicting HAZ at 8 years**										
**Proteins**	**Model 1**		**Model 2**		**Model 3**		**Model 4**		**Model 5**	
**Model Adjusted R-Squared**	**0.0108**		**0.0161**		**0.0461**		**0.0518**		**0.0625**	
	**β**	***p*-Value**	**β**	***p*-Value**	**β**	***p*-Value**	**β**	***p*-Value**	**β**	***p*-Value**
Proteins	0.03	<0.001	0.03	<0.001	0.02	<0.001	0.004	0.625	0.01	0.493
Individual Variable										
Female			0.15	0.002	0.14	0.003	0.14	0.004	0.13	0.006
Community Variable										
Rural/Urban Status					−0.35	<0.001	−0.33	<0.001	−0.21	<0.001
Household Variable										
Total Food Expenditures							0.01	<0.001	0.01	0.025
Maternal schooling										
No Schooling									Reference	
1–6 years									0.14	0.017
7–12 years									0.21	0.016
12+ years									−0.02	0.928
Paternal schooling										
No Schooling									Reference	
1–6 years									0.15	0.014
7–12 years									0.19	0.016
12+ years									0.27	0.029
**Ethiopia—Expenditures at 8 years Predicting HAZ at 12 years**										
**Proteins**	**Model 1**		**Model 2**		**Model 3**		**Model 4**		**Model 5**	
**Model Adjusted R-Squared**	**0.0035**		**0.0029**		**0.0476**		**0.0511**		**0.0579**	
	**β**	***p*-Value**	**β**	***p*-Value**	**β**	***p*-Value**	**β**	***p*-Value**	**β**	***p*-Value**
Proteins	0.02	0.009	0.02	0.009	0.01	0.206	−0.01	0.393	−0.01	0.365
Individual Variable										
Female			0.001	0.983	−0.01	0.776	−0.01	0.833	−0.02	0.689
Community Variable										
Rural/Urban Status					−0.42	<0.001	−0.41	<0.001	−0.30	<0.001
Household Variable										
Total Food Expenditures							0.01	0.007	0.01	0.052
Maternal schooling										
No Schooling									Reference	
1–6 years									0.15	0.008
7–12 years									0.18	0.032
12+ years									0.14	0.466
Paternal schooling										
No Schooling									Reference	
1–6 years									0.09	0.122
7–12 years									0.13	0.105
12+ years									0.16	0.188

Table S3. Unadjusted and Adjusted Models for Predicting Child HAZ Using Past Household Micronutrients Expenditures in Ethiopia.

**Ethiopia—Expenditures at 5 years Predicting HAZ at 8 years**										
**Micronutrients**	**Model 1**		**Model 2**		**Model 3**		**Model 4**		**Model 5**	
**Model Adjusted R-Squared**	**0.0128**		**0.0180**		**0.0470**		**0.0520**		**0.0626**	
	**β**	***p*-Value**	**β**	***p*-Value**	**β**	***p*-Value**	**β**	***p*-Value**	**β**	***p*-Value**
Micronutrients	0.03	<0.001	0.03	<0.001	0.02	<0.001	0.01	0.470	0.01	0.399
Individual Variable										
Female			0.15	0.002	0.14	0.003	0.14	0.003	0.13	0.006
Community Variable										
Rural/Urban Status					−0.34	<0.001	−0.33	<0.001	−0.20	<0.001
Household Variable										
Total Food Expenditures							0.01	0.002	0.01	0.037
Maternal schooling										
No Schooling									Reference	
1–6 years									0.14	0.018
7–12 years									0.21	0.016
12+ years									−0.02	0.915
Paternal schooling										
No Schooling									Reference	
1–6 years									0.15	0.014
7–12 years									0.19	0.016
12+ years									0.27	0.030
**Ethiopia—Expenditures at 8 years Predicting HAZ at 12 years**										
**Micronutrients**	**Model 1**		**Model 2**		**Model 3**		**Model 4**		**Model 5**	
**Model Adjusted R-Squared**	**0.0055**		**0.0049**		**0.0483**		**0.0508**		**0.0576**	
	**β**	***p*-Value**	**β**	***p*-Value**	**β**	***p*-Value**	**β**	***p*-Value**	**β**	***p*-Value**
Micronutrients	0.02	0.001	0.02	0.001	0.01	0.095	−0.003	0.702	−0.01	0.562
Individual Variable										
Female			0.002	0.968	−0.01	0.788	0.01	0.833	−0.02	0.690
Community Variable										
Rural/Urban Status					−0.41	<0.001	−0.41	<0.001	−0.3	<0.001
Household Variable										
Total Food Expenditures							0.01	0.020	0.01	0.085
Maternal schooling										
No Schooling									Reference	
1–6 years									0.15	0.008
7–12 years									0.18	0.031
12+ years									0.13	0.478
Paternal schooling										
No Schooling									Reference	
1–6 years									0.09	0.123
7–12 years									0.13	0.109
12+ years									0.16	0.193

Table S4. Unadjusted and Adjusted Models for Predicting Child HAZ Using Past Household ASF Expenditures in Ethiopia.

Ethiopia—Expenditures at 5 years Predicting HAZ at 8 years										
**Animal Source Foods**	**Model 1**		**Model 2**		**Model 3**		**Model 4**		**Model 5**	
**Model Adjusted R-Squared**	**0.0049**		**0.0099**		**0.0417**		**0.0518**		**0.0622**	
	**β**	***p*-Value**	**β**	***p*-Value**	**β**	***p*-Value**	**β**	***p*-Value**	**β**	***p*-Value**
Animal Source Foods	0.02	0.002	0.02	0.001	0.02	0.009	−0.005	0.608	−0.002	0.834
Individual Variable										
Female			0.15	0.002	0.13	0.004	0.13	0.004	0.13	0.007
Community Variable										
Rural/Urban Status					−0.36	<0.001	−0.33	<0.001	−0.20	<0.001
Household Variable										
Total Food Expenditures							0.01	<0.001	0.01	0.002
Maternal schooling										
No Schooling									Reference	
1–6 years									0.14	0.016
7–12 years									0.21	0.015
12+ years									−0.005	0.981
Paternal schooling										
No Schooling									Reference	
1–6 years									0.15	0.014
7–12 years									0.19	0.015
12+ years									0.28	0.027
**Ethiopia—Expenditures at 8 years Predicting HAZ at 12 years**										
**Animal Source Foods**	**Model 1**		**Model 2**		**Model 3**		**Model 4**		**Model 5**	
**Model Adjusted R-Squared**	**0.0039**		**0.003**		**0.0476**		**0.0508**		**0.0575**	
	**β**	***p*-Value**	**β**	***p*-Value**	**β**	***p*-Value**	**β**	***p*-Value**	**β**	***p*-Value**
Animal Source Foods	0.02	0.006	0.02	0.006	0.01	0.207	−0.004	0.624	−0.005	0.6236
Individual Variable										
Female			−0.0005	0.991	−0.01	0.764	−0.01	0.838	−0.02	0.694
Community Variable										
Rural/Urban Status					−0.42	<0.001	−0.41	<0.001	−0.3	<0.001
Household Variable										
Total Food Expenditures							0.01	0.010	0.005	0.079
Maternal schooling										
No Schooling									Reference	
1–6 years									0.15	0.008
7–12 years									0.18	0.031
12+ years									0.13	0.477
Paternal schooling										
No Schooling									Reference	
1–6 years									0.09	0.124
7–12 years									0.13	0.111
12+ years									0.16	0.198

Table S5. Unadjusted and Adjusted Models for Predicting Child HAZ Using Past Household Starches Expenditures in India.

**India—Expenditures at 5 years Predicting HAZ at 8 years**										
**Starches**	**Model 1**		**Model 2**		**Model 3**		**Model 4**		**Model 5**	
**Model Adjusted R-Squared**	**−0.0006**		**0.0030**		**0.0819**		**0.0831**		**0.1193**	
	**β**	***p*-Value**	**β**	***p*-Value**	**β**	***p*-Value**	**β**	***p*-Value**	**β**	***p*-Value**
Starches	0.0001	0.982	−0.0001	0.990	0.01	0.022	0.004	0.570	0.01	0.224
Individual Variable										
Female			0.12	0.007	0.13	0.003	0.13	0.004	0.12	0.006
Community Variable										
Rural/Urban Status					−0.63	<0.001	−0.62	<0.001	−0.41	<0.001
Household Variable										
Total Food Expenditures							0.005	0.075	0.001	0.643
Maternal schooling										
No Schooling									Reference	
1–6 years									0.17	0.004
7–12 years									0.23	<0.001
12+ years									0.62	<0.001
Paternal schooling										
No Schooling									Reference	
1–6 years									0.11	0.078
7–12 years									0.19	0.002
12+ years									0.38	<0.001
**India—Expenditures at 8 years Predicting HAZ at 12 years**										
**Starches**	**Model 1**		**Model 2**		**Model 3**		**Model 4**		**Model 5**	
**Model Adjusted R-Squared**	**0.0061**		**0.0060**		**0.0645**		**0.0678**		**0.0983**	
	**β**	***p*-Value**	**β**	***p*-Value**	**β**	***p*-Value**	**β**	***p*-Value**	**β**	***p*-Value**
Starches	0.02	<0.001	0.02	<0.001	0.02	0.003	0.002	0.763	0.002	0.744
Individual Variable										
Female			0.04	0.358	0.05	0.304	0.05	0.290	0.04	0.403
Community Variable										
Rural/Urban Status					−0.54	<0.001	−0.53	<0.001	−0.34	<0.001
Household Variable										
Total Food Expenditures							0.01	0.007	0.004	0.114
Maternal schooling										
No Schooling									Reference	
1–6 years									0.11	0.066
7–12 years									0.14	0.044
12+ years									0.49	0.003
Paternal schooling										
No Schooling									Reference	
1–6 years									0.18	0.003
7–12 years									0.30	<0.001
12+ years									0.42	<0.001

Table S6. Unadjusted and Adjusted Models for Predicting Child HAZ Using Past Household Protein Expenditures in India.

**India—Expenditures at 5 years Predicting HAZ at 8 years**										
**Proteins**	**Model 1**		**Model 2**		**Model 3**		**Model 4**		**Model 5**	
**Model Adjusted R-Squared**	**0.0232**		**0.0265**		**0.0943**		**0.0943**		**0.1230**	
	**β**	***p*-Value**	**β**	***p*-Value**	**β**	***p*-Value**	**β**	***p*-Value**	**β**	***p*-Value**
Proteins	0.04	<0.001	0.04	<0.001	0.03	<0.001	0.04	<0.001	0.02	0.003
Individual Variable										
Female			0.12	0.008	0.13	0.004	0.13	0.003	0.12	0.005
Community Variable										
Rural/Urban Status					−0.58	<0.001	−0.57	<0.001	−0.39	<0.001
Household Variable										
Total Food Expenditures							−0.002	0.335	−0.002	0.496
Maternal schooling										
No Schooling									Reference	
1–6 years									0.15	0.012
7–12 years									0.20	0.002
12+ years									0.56	<0.001
Paternal schooling										
No Schooling									Reference	
1–6 years									0.10	0.084
7–12 years									0.18	0.003
12+ years									0.28	<0.001
**India—Expenditures at 8 years Predicting HAZ at 12 years**										
**Proteins**	**Model 1**		**Model 2**		**Model 3**		**Model 4**		**Model 5**	
**Model Adjusted R-Squared**	**0.0219**		**0.0220**		**0.0702**		**0.0701**		**0.0987**	
	**β**	***p*-Value**	**β**	***p*-Value**	**β**	***p*-Value**	**β**	***p*-Value**	**β**	***p*-Value**
Proteins	0.04	<0.001	0.04	<0.001	0.03	<0.001	0.02	0.035	0.01	0.347
Individual Variable										
Female			0.05	0.297	0.05	0.270	0.05	0.272	0.04	0.390
Community Variable										
Rural/Urban Status					−0.50	<0.001	−0.51	<0.001	−0.34	<0.001
Household Variable										
Total Food Expenditures							0.003	0.357	0.002	0.378
Maternal schooling										
No Schooling									Reference	
1–6 years									0.11	0.080
7–12 years									0.13	0.055
12+ years									0.48	0.004
Paternal schooling										
No Schooling									Reference	
1–6 years									0.18	0.004
7–12 years									0.29	<0.001
12+ years									0.41	<0.001

Table S7. Unadjusted and Adjusted Models for Predicting Child HAZ Using Past Household Micronutrients Expenditures in India.

**India—Expenditures at 5 years Predicting HAZ at 8 years**										
**Micronutrients**	**Model 1**		**Model 2**		**Model 3**		**Model 4**		**Model 5**	
**Model Adjusted R-Squared**	**0.0182**		**0.0218**		**0.0906**		**0.0906**		**0.1210**	
	**β**	***p*-Value**	**β**	***p*-Value**	**β**	***p*-Value**	**β**	***p*-Value**	**β**	***p*-Value**
Micronutrients	0.03	<0.001	0.03	<0.001	0.02	<0.001	0.03	<0.001	0.02	0.028
Individual Variable										
Female			0.12	0.007	0.13	0.003	0.13	0.003	0.12	0.005
Community Variable										
Rural/Urban Status					−0.58	<0.001	−0.57	<0.001	−0.39	<0.001
Household Variable										
Total Food Expenditures							−0.003	0.351	−0.001	0.630
Maternal schooling										
No Schooling									Reference	
1–6 years									0.16	0.007
7–12 years									0.21	0.001
12+ years									0.58	<0.001
Paternal schooling										
No Schooling									Reference	
1–6 years									0.10	0.081
7–12 years									0.18	0.003
12+ years									0.37	<0.001

**India—Expenditures at 8 years Predicting HAZ at 12 years**										
**Micronutrients**	**Model 1**		**Model 2**		**Model 3**		**Model 4**		**Model 5**	
**Model Adjusted R-Squared**	**0.0213**		**0.0212**		**0.0702**		**0.02**		**0.0985**	
	**β**	***p*-Value**	**β**	***p*-Value**	**β**	***p*-Value**	**β**	***p*-Value**	**β**	***p*-Value**
Micronutrients	0.03	<0.001	0.03	<0.001	0.02	<0.001	0.02	0.051	0.01	0.421
Individual Variable										
Female			0.04	0.353	0.05	0.307	0.05	0.302	0.04	0.407
Community Variable										
Rural/Urban Status					−0.51	<0.001	−0.51	<0.001	−0.34	<0.001
Household Variable										
Total Food Expenditures							0.002	0.596	0.003	0.488
Maternal schooling										
No Schooling									Reference	
1–6 years									0.11	0.072
7–12 years									0.13	0.055
12+ years									0.48	0.004
Paternal schooling										
No Schooling									Reference	
1–6 years									0.18	0.004
7–12 years									0.29	<0.001
12+ years									0.42	<0.001

Table S8. Unadjusted and Adjusted Models for Predicting Child HAZ Using Past Household ASF Expenditures in India.

India—Expenditures at 5 years Predicting HAZ at 8 years										
**Animal Source Foods**	**Model 1**		**Model 2**		**Model 3**		**Model 4**		**Model 5**	
**Model Adjusted R-Squared**	**0.0281**		**0.0314**		**0.0941**		**0.0938**		**0.1231**	
	**β**	***p*-Value**	**β**	***p*-Value**	**β**	***p*-Value**	**β**	***p*-Value**	**β**	***p*-Value**
Animal Source Foods	0.05	<0.001	0.05	<0.001	0.04	<0.001	0.04	<0.001	0.03	0.003
Individual Variable										
Female			0.12	0.008	0.13	0.004	0.13	0.003	0.12	0.005
Community Variable										
Rural/Urban Status					−0.56	<0.001	−0.55	<0.001	−0.38	<0.001
Household Variable										
Total Food Expenditures							−0.002	0.505	−0.001	0.586
Maternal schooling										
No Schooling									Reference	
1–6 years									0.15	0.011
7–12 years									0.20	0.002
12+ years									0.56	<0.001
Paternal schooling										
No Schooling									Reference	
1–6 years									0.11	0.078
7–12 years									0.18	0.003
12+ years									0.38	<0.001
**India—Expenditures at 8 years Predicting HAZ at 12 years**										
**Animal Source Foods**	**Model 1**		**Model 2**		**Model 3**		**Model 4**		**Model 5**	
**Model Adjusted R-Squared**	**0.0276**		**0.0277**		**0.0722**		**0.072**		**0.0997**	
	**β**	***p*-Value**	**β**	***p*-Value**	**β**	***p*-Value**	**β**	***p*-Value**	**β**	***p*-Value**
Animal Source Foods	0.05	<0.001	0.05	<0.001	0.03	<0.001	0.03	0.005	0.02	0.091
Individual Variable										
Female			0.05	0.272	0.05	0.256	0.05	0.258	0.04	0.373
Community Variable										
Rural/Urban Status					−0.49	<0.001	−0.49	<0.001	−0.33	<0.001
Household Variable										
Total Food Expenditures							0.002	0.439	0.002	0.577
Maternal schooling										
No Schooling									Reference	
1–6 years									0.10	0.093
7–12 years									0.12	0.065
12+ years									0.47	0.004
Paternal schooling										
No Schooling									Reference	
1–6 years									0.18	0.004
7–12 years									0.29	<0.001
12+ years									0.41	<0.001

Table S9. Unadjusted and Adjusted Models for Predicting Child HAZ Using Past Household Starches Expenditures in Peru.

**Peru—Expenditures at 5 years Predicting HAZ at 8 years**										
**Starches**	**Model 1**		**Model 2**		**Model 3**		**Model 4**		**Model 5**	
**Model Adjusted R-Squared**	**−0.0005**		**−0.001**		**0.1543**		**0.1661**		**0.2192**	
	**β**	***p*-Value**	**β**	***p*-Value**	**β**	***p*-Value**	**β**	***p*-Value**	**β**	***p*-Value**
Starches	−0.001	0.785	−0.001	0.775	0.002	0.647	−0.01	0.004	−0.01	0.050
Individual Variable										
Female			0.02	0.713	0.04	0.384	0.04	0.384	0.04	0.474
Community Variable										
Rural/Urban Status					−0.79	<0.001	−0.71	<0.001	−0.44	<0.001
Household Variable										
Total Food Expenditures							0.01	<0.001	0.002	0.095
Maternal schooling										
No Schooling									Reference	
1–6 years									0.26	0.003
7–12 years									0.64	<0.001
12+ years									0.82	<0.001
Paternal schooling										
No Schooling									Reference	
1–6 years									0.10	0.595
7–12 years									0.19	0.327
12+ years									0.33	0.101
**Peru—Expenditures at 8 years Predicting HAZ at 12 years**										
**Starches**	**Model 1**		**Model 2**		**Model 3**		**Model 4**		**Model 5**	
**Model Adjusted R-Squared**	**0.0023**		**0.0024**		**0.1125**		**0.1606**		**0.2086**	
	**β**	***p*-Value**	**β**	***p*-Value**	**β**	***p*-Value**	**β**	***p*-Value**	**β**	***p*-Value**
Starches	−0.01	0.027	−0.01	0.029	0.002	0.556	−0.03	<0.001	−0.02	<0.001
Individual Variable										
Female			−0.06	0.279	−0.04	0.368	−0.05	0.285	−0.06	0.235
Community Variable										
Rural/Urban Status					−0.81	<0.001	−0.68	<0.001	−0.40	<0.001
Household Variable										
Total Food Expenditures							0.01	<0.001	0.01	<0.001
Maternal schooling										
No Schooling									Reference	
1–6 years									0.27	0.005
7–12 years									0.60	<0.001
12+ years									0.70	<0.001
Paternal schooling										
No Schooling									Reference	
1–6 years									0.02	0.921
7–12 years									0.16	0.443
12+ years									0.36	0.101

Table S10. Unadjusted and Adjusted Models for Predicting Child HAZ Using Past Household Protein Expenditures in Peru.

**Peru—Expenditures at 5 years Predicting HAZ at 8 years**										
**Proteins**	**Model 1**		**Model 2**		**Model 3**		**Model 4**		**Model 5**	
**Model Adjusted R-Squared**	**0.0544**		**0.0538**		**0.1707**		**0.1710**		**0.2210**	
	**β**	***p*-Value**	**β**	***p*-Value**	**β**	***p*-Value**	**β**	***p*-Value**	**β**	***p*-Value**
Proteins	0.02	<0.001	0.02	<0.001	0.01	<0.001	0.02	<0.001	0.01	0.006
Individual Variable										
Female			−0.003	0.947	0.03	0.561	0.02	0.594	0.02	0.624
Community Variable										
Rural/Urban Status					−0.71	<0.001	−0.71	<0.001	−0.45	<0.001
Household Variable										
Total Food Expenditures							−0.002	0.203	−0.003	0.062
Maternal schooling										
No Schooling									Reference	
1–6 years									0.27	0.002
7–12 years									0.64	<0.001
12+ years									0.81	<0.001
Paternal schooling										
No Schooling									Reference	
1–6 years									0.10	0.592
7–12 years									0.19	0.335
12+ years									0.32	0.119
**Peru—Expenditures at 8 years Predicting HAZ at 12 years**										
**Proteins**	**Model 1**		**Model 2**		**Model 3**		**Model 4**		**Model 5**	
**Model Adjusted R-Squared**	**0.0506**		**0.0508**		**0.1531**		**0.1534**		**0.2044**	
	**β**	***p*-Value**	**β**	***p*-Value**	**β**	***p*-Value**	**β**	***p*-Value**	**β**	***p*-Value**
Proteins	0.02	<0.001	0.02	<0.001	0.02	<0.001	0.02	<0.001	0.01	<0.001
Individual Variable										
Female			−0.06	0.228	−0.05	0.332	−0.05	0.310	−0.05	0.264
Community Variable										
Rural/Urban Status					−0.77	<0.001	−0.77	<0.001	−0.45	<0.001
Household Variable										
Total Food Expenditures							0.002	0.200	0.0002	0.912
Maternal schooling										
No Schooling									Reference	
1–6 years									0.27	0.005
7–12 years									0.62	<0.001
12+ years									0.71	<0.001
Paternal schooling										
No Schooling									Reference	
1–6 years									0.02	0.938
7–12 years									0.17	0.422
12+ years									0.35	0.110

Table S11. Unadjusted and Adjusted Models for Predicting Child HAZ Using Past Household Micronutrients Expenditures in Peru.

**Peru—Expenditures at 5 years Predicting HAZ at 8 years**										
**Micronutrients**	**Model 1**		**Model 2**		**Model 3**		**Model 4**		**Model 5**	
**Model Adjusted R-Squared**	**0.0569**		**0.0564**		**0.1712**		**0.1721**		**0.2211**	
	**β**	***p*-Value**	**β**	***p*-Value**	**β**	***p*-Value**	**β**	***p*-Value**	**β**	***p*-Value**
Micronutrients	0.02	<0.001	0.02	<0.001	0.01	<0.001	0.01	<0.001	0.01	0.006
Individual Variable										
Female			−0.003	0.946	0.03	0.561	0.02	0.611	0.02	0.634
Community Variable										
Rural/Urban Status					−0.71	<0.001	−0.71	<0.001	−0.44	<0.001
Household Variable										
Total Food Expenditures							−0.002	0.083	−0.003	0.047
Maternal schooling										
No Schooling									Reference	
1–6 years									0.27	0.002
7–12 years									0.64	<0.001
12+ years									0.81	<0.001
Paternal schooling										
No Schooling									Reference	
1–6 years									0.10	0.593
7–12 years									0.18	0.345
12+ years									0.31	0.128
**Peru—Expenditures at 8 years Predicting HAZ at 12 years**										
**Micronutrients**	**Model 1**		**Model 2**		**Model 3**		**Model 4**		**Model 5**	
**Model Adjusted R-Squared**	**0.0538**		**0.0540**		**0.1554**		**0.1551**		**0.2048**	
	**β**	***p*-Value**	**β**	***p*-Value**	**β**	***p*-Value**	**β**	***p*-Value**	**β**	***p*-Value**
Micronutrients	0.02	<0.001	0.02	<0.001	0.02	<0.001	0.02	<0.001	0.01	<0.001
Individual Variable										
Female			−0.06	0.223	−0.05	0.325	−0.05	0.316	−0.05	0.26
Community Variable										
Rural/Urban Status					−0.77	<0.001	−0.77	<0.001	0.45	<0.001
Household Variable										
Total Food Expenditures							0.001	0.567	−0.0004	0.809
Maternal schooling										
No Schooling									Reference	
1–6 years									0.27	0.005
7–12 years									0.62	<0.001
12+ years									0.71	<0.001
Paternal schooling										
No Schooling									Reference	
1–6 years									0.01	0.944
7–12 years									0.17	0.432
12+ years									0.25	0.117

Table S12. Unadjusted and Adjusted Models for Predicting Child HAZ Using Past Household ASF Expenditures in Peru.

**Peru—Expenditures at 5 years Predicting HAZ at 8 years**										
**Animal Source Foods**	**Model 1**		**Model 2**		**Model 3**		**Model 4**		**Model 5**	
**Model Adjusted R-Squared**	**0.0582**		**0.0576**		**0.1710**		**0.1712**		**0.2210**	
	**β**	***p*-Value**	**β**	***p*-Value**	**β**	***p*-Value**	**β**	***p*-Value**	**β**	***p*-Value**
Animal Source Foods	0.02	<0.001	0.02	<0.001	0.01	<0.001	0.02	<0.001	0.01	0.006
Individual Variable										
Female			−0.003	0.954	0.03	0.556	0.02	0.5842	0.02	0.619
Community Variable										
Rural/Urban Status					−0.71	<0.001	−0.71	<0.001	−0.44	<0.001
Household Variable										
Total Food Expenditures							−0.002	0.226	−0.003	0.068
Maternal schooling										
No Schooling									Reference	
1–6 years									0.27	0.002
7–12 years									0.64	<0.001
12+ years									0.81	<0.001
Paternal schooling										
No Schooling									Reference	
1–6 years									0.11	0.577
7–12 years									0.19	0.327
12+ years									0.32	0.117
**Peru—Expenditures at 8 years Predicting HAZ at 12 years**										
**Animal Source Foods**	**Model 1**		**Model 2**		**Model 3**		**Model 4**		**Model 5**	
**Model Adjusted R-Squared**	**0.0580**		**0.0582**		**0.1558**		**0.1558**		**0.2052**	
	**β**	***p*-Value**	**β**	***p*-Value**	**β**	***p*-Value**	**β**	***p*-Value**	**β**	***p*-Value**
Animal Source Foods	0.02	<0.001	0.02	<0.001	0.02	<0.001	0.02	<0.001	0.01	<0.001
Individual Variable										
Female			−0.06	0.238	−0.04	0.344	−0.05	0.326	−0.05	0.272
Community Variable										
Rural/Urban Status					−0.75	<0.001	−0.76	<0.001	−0.45	<0.001
Household Variable										
Total Food Expenditures							0.001	0.327	−0.000003	0.999
Maternal schooling										
No Schooling									Reference	
1–6 years									0.27	0.005
7–12 years									0.62	<0.001
12+ years									0.71	<0.001
Paternal schooling										
No Schooling									Reference	
1–6 years									0.02	0.926
7–12 years									0.17	0.424
12+ years									0.35	0.113

Table S13. Unadjusted and Adjusted Models for Predicting Child HAZ Using Past Household Fats Expenditures in Vietnam.

Vietnam—Expenditures at 5 years Predicting HAZ at 8 years										
**Fats**	**Model 1**		**Model 2**		**Model 3**		**Model 4**		**Model 5**	
**Model Adjusted R-Squared**	**0.0172**		**0.0185**		**0.0881**		**0.1317**		**0.1985**	
	**β**	***p*-Value**	**β**	***p*-Value**	**β**	***p*-Value**	**β**	***p*-Value**	**β**	***p*-Value**
Fats	0.19	<0.001	0.19	<0.001	0.11	0.003	0.001	0.972	−0.03	0.385
Individual Variable										
Female			0.09	0.074	0.10	0.039	0.10	0.036	0.09	0.053
Community Variable										
Rural/Urban Status					−0.7	<0.001	−0.51	<0.001	−0.40	<0.001
Household Variable										
Total Food Expenditures							0.01	<0.001	0.01	<0.001
Maternal schooling										
No Schooling									Reference	
1–6 years									0.57	<0.001
7–12 years									0.66	<0.001
12+ years									0.99	<0.001
Paternal schooling										
No Schooling									Reference	
1–6 years									0.33	0.002
7–12 years									0.39	<0.001
12+ years									0.51	<0.001
**Vietnam—Expenditures at 8 years Predicting HAZ at 12 years**										
**Fats**	**Model 1**		**Model 2**		**Model 3**		**Model 4**		**Model 5**	
**Model Adjusted R-Squared**	**0.003**		**0.0024**		**0.0715**		**0.1102**		**0.1735**	
	**β**	***p*-Value**	**β**	***p*-Value**	**β**	***p*-Value**	**β**	***p*-Value**	**β**	***p*-Value**
Fats	0.06	0.015	0.06	0.015	0.04	0.106	−0.03	0.283	−0.02	0.307
Individual Variable										
Female			0.01	0.846	0.02	0.696	0.01	0.861	0.005	0.921
Community Variable										
Rural/Urban Status					−0.75	<0.001	−0.46	<0.001	−0.35	<0.001
Household Variable										
Total Food Expenditures							0.01	<0.001	0.01	<0.001
Maternal schooling										
No Schooling									Reference	
1–6 years									0.66	<0.001
7–12 years									0.69	<0.001
12+ years									0.95	<0.001
Paternal schooling										
No Schooling									Reference	
1–6 years									0.24	0.041
7–12 years									0.39	0.001
12+ years									0.57	<0.001

Table S14. Unadjusted and Adjusted Models for Predicting Child HAZ Using Past Household Proteins Expenditures in Vietnam.

Vietnam—Expenditures at 5 years Predicting HAZ at 8 years										
**Proteins**	**Model 1**		**Model 2**		**Model 3**		**Model 4**		**Model 5**	
**Model Adjusted R-Squared**	**0.0925**		**0.0932**		**0.1277**		**0.1338**		**0.1984**	
	**β**	***p*-Value**	**β**	***p*-Value**	**β**	***p*-Value**	**β**	***p*-Value**	**β**	***p*-Value**
Proteins	0.03	<0.001	0.03	<0.001	0.02	<0.001	0.01	0.048	0.003	0.477
Individual Variable										
Female			0.07	0.138	0.08	0.078	0.09	0.050	0.08	0.062
Community Variable										
Rural/Urban Status					−0.52	<0.001	−0.50	<0.001	−0.39	<0.001
Household Variable										
Total Food Expenditures							0.01	<0.001	0.01	0.007
Maternal schooling										
No Schooling									Reference	
1–6 years									0.57	<0.001
7–12 years									0.66	<0.001
12+ years									0.98	<0.001
Paternal schooling										
No Schooling									Reference	
1–6 years									0.32	0.003
7–12 years									0.38	<0.001
12+ years									0.50	<0.001
**Vietnam—Expenditures at 8 years Predicting HAZ at 12 years**										
**Proteins**	**Model 1**		**Model 2**		**Model 3**		**Model 4**		**Model 5**	
**Model Adjusted R-Squared**	**0.0822**		**0.0816**		**0.1110**		**0.114**		**0.1730**	
	**β**	***p*-Value**	**β**	***p*-Value**	**β**	***p*-Value**	**β**	***p*-Value**	**β**	***p*-Value**
Proteins	0.03	<0.001	0.03	<0.001	0.02	<0.001	0.01	0.007	0.001	0.812
Individual Variable										
Female			0.005	0.926	0.01	0.797	0.01	0.844	0.005	0.924
Community Variable										
Rural/Urban Status					−0.53	<0.001	−0.47	<0.001	−0.36	<0.001
Household Variable										
Total Food Expenditures							0.005	0.016	0.01	0.006
Maternal schooling										
No Schooling									Reference	
1–6 years									0.67	<0.001
7–12 years									0.69	<0.001
12+ years									0.95	<0.001
Paternal schooling										
No Schooling									Reference	
1–6 years									0.24	0.042
7–12 years									0.39	0.002
12+ years									0.57	<0.001

Table S15. Unadjusted and Adjusted Models for Predicting Child HAZ Using Past Household Micronutrients Expenditures in Vietnam.

**Vietnam—Expenditures at 5 years Predicting HAZ at 8 years**										
**Micronutrients**	**Model 1**		**Model 2**		**Model 3**		**Model 4**		**Model 5**	
**Model Adjusted R-Squared**	**0.097**		**0.0977**		**0.1311**		**0.1346**		**0.1984**	
	**β**	***p*-Value**	**β**	***p*-Value**	**β**	***p*-Value**	**β**	***p*-Value**	**β**	***p*-Value**
Micronutrients	0.02	<0.001	0.02	<0.001	0.02	<0.001	0.01	0.021	0.003	0.462
Individual Variable										
Female			0.07	0.120	0.08	0.07	0.09	0.051	0.08	0.061
Community Variable										
Rural/Urban Status					−0.51	<0.001	−0.50	<0.001	−0.39	<0.001
Household Variable										
Total Food Expenditures							0.01	0.007	0.01	0.017
Maternal schooling										
No Schooling									Reference	
1–6 years									0.57	<0.001
7–12 years									0.65	<0.001
12+ years									0.98	<0.001
Paternal schooling										
No Schooling									Reference	
1–6 years									0.32	0.003
7–12 years									0.38	<0.001
12+ years									0.50	<0.001
**Vietnam—Expenditures at 8 years Predicting HAZ at 12 years**										
**Micronutrients**	**Model 1**		**Model 2**		**Model 3**		**Model 4**		**Model 5**	
**Model Adjusted R-Squared**	**0.0856**		**0.0851**		**0.1114**		**0.113**		**0.1729**	
	**β**	***p*-Value**	**β**	***p*-Value**	**β**	***p*-Value**	**β**	***p*-Value**	**β**	***p*-Value**
Micronutrients	0.02	<0.001	0.02	<0.001	0.02	<0.001	0.01	0.012	0.0001	0.979
Individual Variable										
Female			0.004	0.932	0.01	0.805	0.01	0.844	0.005	0.9277
Community Variable										
Rural/Urban Status					−0.51	<0.001	−0.47	<0.001	−0.36	<0.001
Household Variable										
Total Food Expenditures							0.004	0.045	0.01	0.008
Maternal schooling										
No Schooling									Reference	
1–6 years									0.67	<0.001
7–12 years									0.70	<0.001
12+ years									0.95	<0.001
Paternal schooling										
No Schooling									Reference	
1–6 years									0.24	0.041
7–12 years									0.39	0.002
12+ years									0.58	<0.001

Table S16. Unadjusted and Adjusted Models for Predicting Child HAZ Using Past Household ASF Expenditures in Vietnam.

**Vietnam—Expenditures at 5 years Predicting HAZ at 8 years**										
**Animal Source Foods**	**Model 1**		**Model 2**		**Model 3**		**Model 4**		**Model 5**	
**Model Adjusted R-Squared**	**0.0923**		**0.0930**		**0.1277**		**0.1338**		**0.1984**	
	**β**	***p*-Value**	**β**	***p*-Value**	**β**	***p*-Value**	**β**	***p*-Value**	**β**	***p*-Value**
Animal Source Foods	0.03	<0.001	0.03	<0.001	0.02	<0.001	0.01	0.045	0.003	0.449
Individual Variable										
Female			0.07	0.141	0.08	0.080	0.09	0.050	0.08	0.062
Community Variable										
Rural/Urban Status					−0.52	<0.001	−0.50	<0.001	−0.39	<0.001
Household Variable										
Total Food Expenditures							0.01	<0.001	0.01	0.007
Maternal schooling										
No Schooling									Reference	
1–6 years									0.57	<0.001
7–12 years									0.66	<0.001
12+ years									0.98	<0.001
Paternal schooling										
No Schooling									Reference	
1–6 years									0.32	0.003
7–12 years									0.38	<0.001
12+ years									0.50	<0.001
**Vietnam—Expenditures at 8 years Predicting HAZ at 12 years**										
**Animal Source Foods**	**Model 1**		**Model 2**		**Model 3**		**Model 4**		**Model 5**	
**Model Adjusted R-Squared**	**0.0818**		**0.0812**		**0.1104**		**0.1134**		**0.1730**	
	**β**	***p*-Value**	**β**	***p*-Value**	**β**	***p*-Value**	**β**	***p*-Value**	**β**	***p*-Value**
Animal Source Foods	0.03	<0.001	0.03	<0.001	0.02	<0.001	0.01	0.008	0.001	0.896
Individual Variable										
Female			0.004	0.941	0.01	0.808	0.01	0.852	0.005	0.926
Community Variable										
Rural/Urban Status					−0.53	<0.001	−0.47	<0.001	−0.36	<0.001
Household Variable										
Total Food Expenditures							0.005	0.011	0.01	0.004
Maternal schooling										
No Schooling									Reference	
1–6 years									0.67	<0.001
7–12 years									0.70	<0.001
12+ years									0.95	<0.001
Paternal schooling										
No Schooling									Reference	
1–6 years									0.24	0.041
7–12 years									0.39	0.002
12+ years									0.58	<0.001

© 2020 by the authors. Submitted for possible open access publication under the terms and conditions of the Creative Commons Attribution (CC BY) license (http://creativecommons.org/licenses/by/4.0/).
